# Design of an improved set of oligonucleotide primers for genotyping MeCP2^tm1.1Bird ^KO mice by PCR

**DOI:** 10.1186/1750-1326-2-16

**Published:** 2007-08-31

**Authors:** Julie Miralvès, Eddy Magdeleine, Etienne Joly

**Affiliations:** 1 Equipe de Neuro-Immuno-Génétique Moléculaire, IPBS, UMR CNRS 5089, 205 route de Narbonne, 31077 Toulouse Cedex, France

## Abstract

**Background:**

The strain of MeCP2^tm1.1Bird ^mice is a broadly used model for Rett syndrome. Because males carrying the invalidated MeCP2 locus are sterile, this strain has to be maintained in a heterozygous state. All animals therefore have to be genotyped at every generation to discriminate those carrying the invalidated allele (+/- females and y/- males) from those that do not. This is conveniently carried out by PCR on tail genomic DNA but because the primer pairs described initially for this purpose yield very similar size DNA bands on the WT and the KO alleles, this requires to carry out two independent PCR reactions on tail DNA preparations from all animals.

**Results:**

After cloning and sequencing the PCR fragment amplified on the KO allele, we tested several sets of primers that were designed to yield PCR fragments of different sizes on the KO and WT alleles.

**Conclusion:**

We have thus identified a set of three primers that allows for efficient genotyping of the animals by a single PCR reaction. Furthermore, using of this set of primers also resolves a recurrent problem related to the tendency of one of the initial primers to give rise to a non specific band because of its capacity to anneal at both ends of a repeated genomic element which we have identified as MurvyLTR.

## Background

Rett syndrome (RTT) is a severe form of mental retardation which affects mostly girls. RTT is caused by dominant spontaneous mutations in the X-linked gene *MECP2 *[1]. Several lines of mice carrying invalidated *Mecp2 *gene have been generated [2-4], which serve as very useful models to decipher the function(s) of MeCP2 and to try to understand the pathogenesis of RTT. Although hemi-zygote male mice carrying an invalidated *Mecp2 *gene (y/-) are viable, they suffer from a more severe form of the disease than the heterozygote females (+/-) and are sterile. Female mice, on the other hand, develop clinical signs later than the males and remain fertile for several months, albeit with a somewhat limited breeding efficiency [5]. Because the males are sterile, one must maintain this mouse strain by crossing heterozygous females with wild type (WT) males. The offspring therefore must be systematically screened by PCR genotyping. A genotyping protocol which was initially devised by Brian Hendrich and Jacky Guy, who also generated these mice [2], is available on the web site of the Jackson Laboratory, where this strain of mice is maintained and distributed to many labs worldwide. This protocol calls for two separate PCR reactions to amplify either a 415 bp fragment on the WT allele with the P5-P7 primer pair, or a 425 bp fragment on the disrupted allele with the P5-P6 primer pair. Because of their very similar sizes, these bands have to be run separately on an agarose gel. The primers we used for our study, which were suggested by the laboratory of Professor Adrian Bird, are longer that those found on the Jackson Laboratory site by a few nucleotides (see materials and methods).

Genotyping of males with this protocol should yield a PCR product with either one pair of primer or the other (P5-P6 for the KO, P5-P7 for the WT). A further unexpected drawback we experienced with the oligonucleotides we used was that genotyping of males frequently gave rise to bands in both sets of PCR reactions, with the unexpected band being slightly more diffuse and higher than the other band. Because the maintenance of these MeCP2 KO mice is already very time consuming and cumbersome, we felt that it would be very useful to be able to genotype the mice via a single PCR reaction. We have therefore devised an optimised genotyping protocol by testing several primer combinations, which is more specific than the original one and yields DNA fragments on the WT and KO alleles that can easily be distinguished in a single lane on an agarose gel.

## Results and Discussion

### Design of the optimised P3 primer set

Although the cloning strategy used to generate the mice is clearly described in the original paper [2], we were unsure of the exact DNA sequence of the disrupted allele. With a view to devise a new set of primers for the genotyping of MeCP2^tm1.1Bird ^mice, we therefore cloned the PCR fragments generated by the P5-P6 and P5-P7 primer pairs into the T overhang pDrive vector (Qiagen) and sequenced them. These DNA sequences (em:AM691835 and em:AM691836), which were extended by a few tens of nucleotides on either side by identifying the corresponding genomic regions by performing Blast searches, were then submitted to the Primer3 and Gene Fisher online WEB servers for the design of optimised PCR primers. We then picked the best hits proposed by each, and ordered all six oligonucleotides (table 1).

**Table 1:** Size of DNA fragments obtained by PCR with the novel oligonucleotides tested in this study.

	P3 RV	GF RV
	CCACCCTCCAGTTTGGTTTA	GTTTTGTTCCCCACCCTCCA
GF KO FW	475	486
ACTTTGTCCTGCTGCCTCCA		
P3 KO FW	458	469
CCATGCGATAAGCTTGATGA		
P3 WT FW	411	421
GACCCCTTGGGACTGAAGTT		
GF WT FW	413	424
TCGGACCCCTTGGGACTGA		

These oligonucleotides were chosen by submitting sequences AM691835 and AM691836 to the P3 [8] and gene Fisher [9] primer-design web servers.

To identify those that worked more efficiently and reliably, these primers were then used with various protocols in all the possible eight combinations. This led us to choose all three primers suggested by the P3 web server. The conditions to perform the diagnostic PCRs in a single tube were then further optimised for variables such as annealing temperature, concentrations of primers, number of cycles and length of elongation time, with a view to obtain bands of comparable intensities on heterozygote females. This finalised protocol is provided in the methods section.

**Figure 1:**
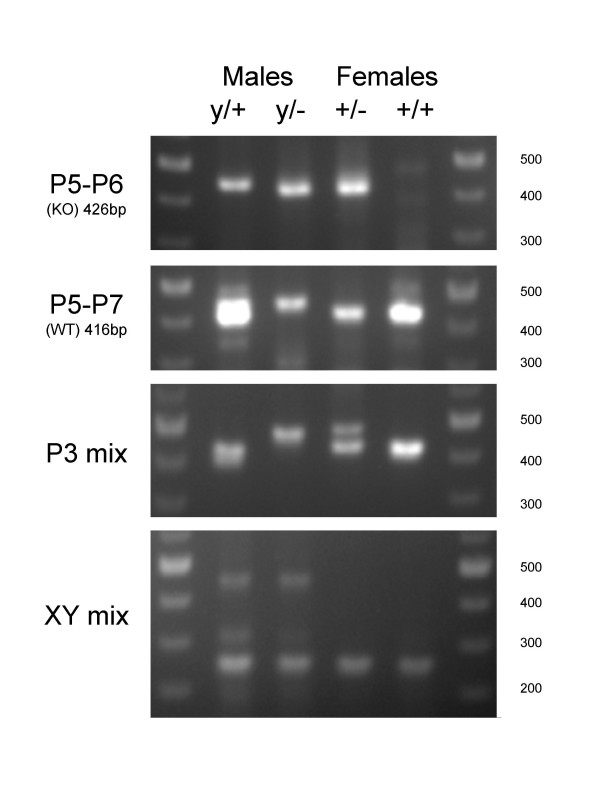
**Electrophoretic analysis of genotyping PCR products**. Genomic tail DNAs were prepared as detailed in the methods sections. 20 ng of the same DNA preparations of the four genotypes (KO male: y/- ; WT male: y/+; Heterozygote female: +/- ; WT female: +/+) were submitted to the different PCR amplification protocols detailed in the methods section, before loading on a 1.5% agarose gel.

The typical result of a 1.5% agarose gel to analyse the PCR reactions obtained with these conditions on all four genotypes (y/- ; y/+; +/- ; +/+) is shown on Fig 1, showing that the 411 bp WT band can be clearly distinguished from the 458 bp KO band, and that both bands are amplified to a comparable extent in heterozygote females. Compared to the results obtained on the same DNAs with the P5-P6 and P5-P7 primer pairs, a further advantage of this set of primers is that at least one band is generated with all four genotypes, thereby cancelling the need to ascertain that an absence of band in a lane is not due to a failed PCR reaction (for this purpose, the Jackson Laboratory recommends using an additional primer pair as an internal control, which amplifies a 312 bp fragment on the IL-2 gene).

### Characterisation of the undesirable 'ghost' bands

As alluded to earlier, we have also found that genotyping of males with the P5-P6 and P5-P7 sets of primers often tends to yield DNA bands in both PCR reactions. In our hands, this has occurred more frequently than one time out of two. When they arise, the non-specific bands are usually slightly higher and sometimes more diffuse than the bona fide bands. An example of such non-specific bands can be seen for the y/+ male with the P5-P6 pair, for the y/- male with the P5-P7 primer pair, and probably contributes to the upper part of the broad and intense band seen in the lane of the y/+ male with the same P5-P7 pair.

Attempts at sequencing these PCR products directly yielded no signal with the P6 or P7 primers, and unreadable sequences with the P5 primer. This observation found its explanation when were carried out secondary PCR amplifications with only one of the three primers in the reaction, starting from 1 μl of the initial PCR, diluted 1000 fold. These PCRs carried out with a single primer consistently yielded high amounts of DNA when they were performed with primer P5, and not with the two others. Six plasmid clones obtained after inserting this PCR fragment into PCR-TOPO were submitted to automated sequencing. This revealed that they all contained the P5 primer at either end, surrounding inserts that were very closely related, but not identical, to one another (95–99 % identity). Blast searches performed on the nr NCBI database identified over one hundred different hits ranging from 99 % to 90 % identity for each of these sequences. Quite remarkably, these hits were almost all BAC sequences mapping to the Y chromosome. This suggested that the sequences carried by the cloned inserts actually corresponded to repetitive genomic DNA sequence present specifically on the mouse Y chromosome. This was confirmed by a search with the CENSOR web server [6], which identified this DNA as a MurvyLTR repetitive element (for MUrine RetroVirus Y). MurvyLTRs are known to be present in dozens of copies on the Y chromosome of certain mouse species and not others [6]. This provides an explanation for the fact that we never obtained this ghost band in the P5-P6 PCRs from WT females.

The homology of the P5 primer with the Y chromosome sequences is, however, sufficiently low to be missed by blast searches either on the whole genome, or simply on the corresponding BAC sequences. Undisrupted pairing of the 3' end of P5 primer to the sequences carried by the Y chromosome BAC sequences identified by blast occurs only over 11 consecutive nucleotides on one side, and 6 or 7 nucleotides on the other (final nucleotides corresponding for example to positions 163994 and 163580 on BAC RP24-233K2 (acc: gb|AC147265.2), to 171119 and 170710 on BAC clone RP24-120L13(acc: gb|AC182036.2), and to 61 and 484 of the MurvyLTR sequence from the Repbase database). The three extra nucleotides (AAG) present at the 3' end of the P5 primer we used compared to the one found in the protocol provided by the Jackson laboratory thus probably contributed very significantly to the undesirable amplification of these 'ghost' bands. Our observations underline a potential problem that can derive from the capacity of certain oligonucleotides to anneal, even imperfectly, to DNA sequences that are repeated many times in the genome.

## Conclusion

We hope that the new set of primers we have designed will prove useful to the many laboratories that work with the strain of MeCP2^tm1.1Bird ^KO mice.

## Methods

### Mice

MeCP2^tm1.1Bird ^+/- female mice were provided by Brian Hendrich from the ISCR, Edinburgh, UK, and mated with C57BL/6 wild-type male mice purchased from the Centre de Recherche et d'Élevage Janvier, France. All experiments involving animals were performed in compliance with the relevant laws and institutional guidelines.

### Tissue Biopsy and DNA extraction

Mice born from MeCP2^tm1.1Bird ^+/- female and C57BL/6 wild-type male mate were weaned at 3 weeks of age, at which times the distal 1 cm of tail was excised with a sterile razor blade. Tail biopsies were placed in 1,5 ml microcentrifuge tubes and digested overnight at 55°C with 600 μl of tail digestion TNES buffer (400 mM NaCl, 100 mM EDTA, 0.6 % SDS, 10 mM Tris, pH 7.5) containing 35 μg proteinase K (Promega). 166,7 μl NaCl 6M were added and samples shaken vigorously for 15 sec prior to 14000 × g centrifugation for 5 min at room temperature. DNA was precipitated from the aqueous phase with one volume of ice-cold 95 % ethanol. Pellets were washed with one volume of 70 % ethanol and rapidly dried at room temperature. The pellets were then dissolved in 100 μl TE buffer, warmed at 65°C for at least 10 min and quantified by OD_260_. When they were not used immediately, samples were stored under ethanol at -20°C.

**Primers: **All primers were ordered from Sigma.

P5 primer : 5'-**T**GGTAAAGACCCATGTGACCC**AAG**-3'

P6 primer : 5'-TCCACCTAGCCTGCCTGTAC**TT**-3'

P7 primer : 5'-GGCTTGCCACATGACAA**GAC**-3'

The residues shown in bold correspond to the additional nucleotides in the primers we used compared to those recommended in the protocol provided on the web site of the Jackson Laboratory.

P3 WT FW : 5'-GACCCCTTGGGACTGAAGTT-3'

P3 KO FW: 5'-CCATGCGATAAGCTTGATGA-3'

P3 RV : 5'-CCACCCTCCAGTTTGGTTTA-3'

GF WT FW : 5'-TCGGACCCCTTGGGACTGA-3'

GF KO FW : 5'-ACTTTGTCCTGCTGCCTCCA-3'

GF RV : 5'-GTTTTGTTCCCCACCCTCCA-3'

The sizes expected from the various combinations of these six primers are listed in table 1.

Sexing primers [7]

*Sry (441 bp*, Y chromosome):

5'-TCATGAGACTGCCAACCACAG-3'

5'-CATGACCACCACCACCACCAA-3'

*Myog (245 bp*, X chromosome):

5'-TTACGTCCATCGTGGACAGC-3',

5'-TGGGCTGGGTGTTAGTCTTA-3'.

### PCR

The PCR reactions were all performed in a final volume of 20 μl containing approximately 20 ng tail DNA. The PCR cocktail was made up to give the following final concentration when added to sample solution: 1X NEB PCR Reaction Buffer, 2,5 mM MgCl_2_, 200 μM dNTPs, 1 μM primers mix and 1 U Taq DNA Polymerase (NEB).

PCR conditions for P5-P6 amplification (with 500 nM of each primer) were as follows: denaturation at 94°C for 2 min followed by 30 cycles of 94°C for 30 s, annealing at 64°C for 30 s and extension at 72°C for 1 min, followed by a final extension step at 72°C for 5 min and the reactions were then held at 4°C until analysis.

PCR conditions for P5-P7 (with 500 nM of each primer) were as follows: denaturation at 94°C for 2 min followed by 30 cycles of 94°C for 30 s, annealing at 60°C for 30 s and extension at 72°C for 30 s, followed by a final extension step at 72°C for 5 min and the reactions were then held at 4°C until analysis.

PCR conditions for XY amplification were identical to those used for the P5-P7 pair, but with 250 nM of each of the four primers.

PCR conditions for P3 amplification (with 500 nM of P3 KO FW, 250 nM of P3 WT FW and 250 nM of P3 RV) were as follows: denaturation at 95°C for 2 min followed by 35 cycles of 95°C for 30 s, annealing at 64°C for 30 s and extension at 72°C for 40 s, followed by a final extension step at 72°C for 7 min and the reactions were then held at 4°C until analysis.

PCR products were all analysed by 1,5 % agarose gel electrophoresis in 1X TAE buffer, stained with ethidium bromide and photographed with Biorad gel doc XR using Quantity One 4.6.2 basic software. The molecular weight marker used was 100 bp DNA Ladder (New England Biolabs Inc).

### PCR product purification, cloning and sequencing

The expected 415 bp P5-P7 and 426 bp P5-P6 amplification products were extracted and purified from a 1,5 % agarose gel using the QIAquick gel extraction kit (Qiagen). They were afterwards cloned into pDrive Cloning vector following the recommended QIAGEN PCR cloning kit protocol and amplified by transformation into competent DH5-α bacteria. The recovered plasmids were then sequenced by the MilleGen company (Toulouse) using the T7 promoter primer.

The unexpected "ghost" P5-P6 or P5-P7 amplification products were re-amplified in secondary PCR reactions containing either both oligonucleotides or just the P5 primer. The resulting bands were then extracted and purified from a 1,5 % agarose gel using the QIAquick gel extraction kit (Qiagen), before cloning into the pCR 2.1-TOPO vector following the instructions of the TOPO TA Cloning kit (Invitrogen) and amplified by transformation into highly efficient TOP10 bacteria (Invitrogen). The recovered plasmids were then sequenced by the MilleGen company (Toulouse) using the M13 reverse primer.

### Software

On-line web servers used in this study: Primer3 [8]; GeneFisher [9]; Blast [10]; CENSOR and Repbase [11].

## Abbreviations

MeCP2- Methyl Cytosine binding Protein 2.

PCR- Polymerase Chain Reaction.

KO- Knock Out (mouse with invalidated gene).

WT- Wild Type.

bp- Base pair.

IL-2- Interleukin 2.

BAC- Bacterial Artificial Chromosome.

G- Guanidine.

C- Cytidine.

LTR- Long Terminal Repeat.

## Competing interests

The author(s) declare that they have no competing interests.

## Authors' contributions

JM and EM performed the experiments, EJ supervised the work and wrote the paper.
